# Trajectories of neutrophil-to-lymphocyte ratios during neoadjuvant chemotherapy correlate with short- and long-term outcomes in gastric cancer: a group-based trajectory analysis

**DOI:** 10.1186/s12885-024-11950-2

**Published:** 2024-02-16

**Authors:** Hua-Long Zheng, Fu-Hai Wang, Ling-Kang Zhang, Ping Li, Chao-Hui Zheng, Qi-Yue Chen, Chang-Ming Huang, Jian-Wei Xie

**Affiliations:** 1 Department of Gastric Surgery, Fujian Medical University Union Hospital, No. 29 Xinquan Road, Fuzhou, Fujian Province 350001 China; 2 Fujian Provincial Minimally Invasive Medical Center, Fuzhou, China; 3 Department of General Surgery, Fujian Medical University Union Hospital, Fuzhou, China

**Keywords:** Gastric cancer, Neoadjuvant chemotherapy, Systemic inflammatory cytokines, Group-based trajectory model, Survival prognosis

## Abstract

**Background:**

Systemic inflammatory factors can predict the survival prognosis of gastric cancer (GC) patients after neoadjuvant chemotherapy (NACT). However, whether longitudinal changes in systemic inflammatory factors are associated with short - and long-term outcomes has not been reported.

**Methods:**

This study is a retrospective analysis of 216 patients with advanced gastric cancer who received NACT between January 2011 and June 2019, comparing receiver operating characteristic (ROC) curves for screening suitable inflammatory markers. Group-based trajectory modeling (GBTM) was used to analyze longitudinal changes in inflammatory markers during NACT to identify different potential subgroups and to compare postoperative complications, recurrence-free survival (RFS), and overall survival (OS) among subgroups.

**Results:**

Ultimately, neutrophil-lymphocyte ratio (NLR) had the highest area under the curve (AUC) value in predicting prognosis was included in the GBTM analysis. Three trajectories of NLR were obtained: Stable group (SG) (*n* = 89), Ascent-descend group (ADG) (*n* = 80) and Continuous descend group (CDG) (*n* = 47). Compared with SG, ADG and CDG are associated with an increased risk of postoperative recurrence and death. The median time of RFS and OS of SG was longer than that of ADG and CDG (median RFS 81 vs. 44 and 22 months; median OS 69 vs. 41 and 30 months). In addition, CDG had significantly higher postoperative serious complications than SG and ADG (17 (36.2%) vs. 17 (19.1%) and 12 (15.0%); *p* = 0.005).

**Conclusion:**

There were different trajectories of NLR during NACT, and these potential trajectories were significantly associated with severe postoperative complications, recurrence, and mortality in patients with GC.

**Supplementary Information:**

The online version contains supplementary material available at 10.1186/s12885-024-11950-2.

## Introduction

Globally, gastric cancer (GC) is the fifth most common cancer and the third leading cause of cancer-related deaths [1]. GC patients are mostly diagnosed at the late stage when they develop symptoms [2, 3]. However, due to atypical symptoms, most GC patients are already in the advanced stage when diagnosed [2, 3]. Although multimodal therapy has been widely used in clinical practice, the survival prognosis of advanced GC patients is still not optimistic [4]. To improve the survival of GC patients, Wilke et al. first proposed neoadjuvant chemotherapy (NACT) in 1989 [5]. Since then, several prospective studies have shown that NACT can improve the prognosis of patients [6–8]. The advantages of NACT include better disease tolerance, tumor downstaging, elimination of occult micrometastases, improving the chance of radical resection and cure of cancer, reducing tumor recurrence, and prolonging survival [9–13]. Therefore, guidelines recommend NACT as the standard treatment for locally advanced gastric cancer [14, 15].

In 1983, Welshaw first revealed the association between inflammation and cancer [16]. In recent years, clinicians have conducted a series of studies on the association between inflammatory markers and malignant tumors. Evidence shows that systemic inflammation is very common in most patients with malignant tumors, and the occurrence and development of inflammatory markers are closely associated with tumor progression and prognosis [17–19]. Several studies have reported that systemic inflammatory markers, such as neutrophil-to-lymphocyte ratio (NLR), platelet-to-lymphocyte ratio (PLR), lymphocyte-to-monocyte ratio (LMR), C-reactive protein/albumin (CRP/Alb) ratio, systemic immune inflammation index (SII) and modified systemic inflammation score (mSIS) have unique prognostic roles in gastric cancer and are of certain value in predicting the clinical prognosis of GC [20–25]. However, previous studies on the relationship between inflammatory markers and the prognosis of GC have mostly focused on the GC population without NACT, and only limited studies have shown that inflammatory markers can be used as independent predictors of the long-term outcomes in GC patients receiving NACT [26–28]. It is worth noting that previous studies on gastric cancer and systemic inflammatory factors were limited to analyzing single-point blood samples before NACT and before and after surgery while ignoring the longitudinal changes in GC inflammatory markers over time. Researchers typically divide the results of a single blood sample into two groups based on cut-off values: high and low, which is equivalent to an intuitive dichotomous comparison of lower inflammatory markers with higher inflammatory markers. Single-point analysis may be affected by other uncontrollable factors, such as the presence of infection foci, myelosuppression after chemotherapy, and artificial injection of colony-stimulating factor, which is a certain chance, and deviate from the experimental accuracy.

Particular trajectory patterns may be closely associated with the prognostic risk of disease [29–32]. Therefore, compared with single-point monitoring of the level of specific inflammatory markers, monitoring its trend may be a better predictor of long-term survival. No studies have shown a relationship between the trajectory of inflammatory markers during NACT and long-term survival outcomes in GC patients receiving NACT. We hypothesized that distinct inflammatory marker trajectories during NACT were associated with long-term overall mortality and tumor recurrence. Group-based trajectory modeling (GBTM) is mainly used to analyze longitudinal data to explore population heterogeneity [33, 34]. GBTM is increasingly used in clinical research to delineate the different developmental processes of events and assess their heterogeneity in clinical outcomes. Therefore, we used GBTM to conduct a retrospective cohort study of a population who underwent radical gastrectomy after NACT to assess the potential association between the trajectories of different longitudinal trends of GC-specific inflammatory markers during NACT and short- and long-term clinical outcomes.

## Methods

### Study design and participants

This is a single-center retrospective cohort study conducted in a tertiary-grade hospital. Patients who underwent radical gastrectomy after NACT between January 2011 and June 2019 were analyzed. The inclusion criteria were as follows: (1) Pathological diagnosis of gastric adenocarcinoma; (2) Received NACT for at least two cycles; (3) no evidence of distant metastasis or para-aortic lymphadenopathy; (4) Underwent radical gastrectomy; (5) At least four blood samples have been tested. The exclusion criteria were as follows: (1) Gastric stump cancer; (2) merging other malignant tumors; (3) Emergency surgery due to gastrointestinal bleeding or perforation; (4) Autoimmune disease or recent steroid use history. A total of 216 patients who met the inclusion criteria were finally included, and the specific inclusion and exclusion process is shown in Figure S1. The institutional review board of the Union Hospital of Fujian Medical University approved this study.

### Data management and definition

All patients’ clinicopathological and follow-up data were retrieved from the gastric cancer database, prospectively collected, and maintained at our center. The traditional key inflammatory markers, including NLR, PLR, NAR, PAR, LMR, and SIS, were included in the discussion and comparison in this study. The calculation formulas of inflammatory markers have been described in many studies (Supplementary Materials for Details) [17–25]. By comparing the area under the curve (AUC) of the receiver operating characteristic (ROC) curve of traditional inflammatory markers predicting 5-year overall survival (5-year OS) and 5-year relapse-free survival (5-year RFS), we found NLR to have a good AUC value and included it in the subsequent trajectory analysis (Supplementary Table S1-2). Blood samples were collected at five time points during the NACT period (each sample was taken on an empty stomach, and the time points were set as the following: the first diagnosis of GC, after the first NACT, after the second NACT, after the third NACT, and one week before surgery, with an interval of 3 weeks ± 1 week at each time point). It is worth noting that if the patient received multiple blood samples within a time point range, the average of the results of multiple blood samples was selected as the NLR value at that time point. Surgery and a lymph node dissection were performed following the guidelines of the Japanese Society for the Study of Gastric Cancer [14]. Tumor staging was performed according to the 8th edition of AJCC for clinical and pathological TNM staging [15]. The pathological response was evaluated by tumor regression grades (TRGs) [35].

### Treatments

In this study, patients received at least two cycles of NACT, with surgery scheduled 3–4 weeks after the end of the last NACT. Under normal circumstances, the surgical method is radical GC resection combined with D2 lymph node dissection. When it is suspected or confirmed that the tumor involves adjacent organs, combined organ resection, including the colon, pancreas, liver, and spleen resection, will be considered. The NACT regimen is based on fluorouracil; the combination drugs include cisplatin, oxaliplatin, loplatin, paclitaxel, and docetaxel (Supplementary Materials for Details). All patients received fluorouracil-based NAC and were routinely recommended to receive AC after surgery, including 2 preoperative and 6 postoperative 3-week cycles of SOX/XELOX (40 to 60 mg/m2 of S-1 or 1000 mg/m2 of capecitabine orally twice daily on days 1 to 14 and 130 mg/m2 of oxaliplatin intravenously on day 1) [36, 37], 3 preoperative and 6 postoperative 2-week cycles of FOLFOX4 (85 mg/m2 of oxaliplatin intravenously on day 1, 200 mg/m2 of folinic acid as a 2-hour intravenous infusion followed by a 400-mg/m2 bolus of fluorouracil, and a 22-hour intravenous infusion of 600 mg/m2 of fluorouracil) [38]. The above chemotherapy regimen and dose have been reported previously [39].

### Evaluation of complications

The complications that occurred during the postoperative hospitalization were recorded. Postoperative complications were evaluated according to the Clavien-Dindo classification (CDC) [40]. Higher grade complications were defined as when patients had two or more complications. The most common postoperative complication in this study was “pulmonary infection.” Other complications included incision infection, abdominal infection, septic shock, anastomotic fistula, pancreatic fistula, intestinal obstruction, secondary bleeding, and others.

### Follow-ups

From the surgery date, each patient was followed regularly until June 2022 or until death. Follow-up was performed every three months for two years and every six months after that until the fifth year. Routine follow-up protocols include physical examination, laboratory testing (including cancer antigens such as CA19-9 and CA72-4, Carcinoembryonic antigen, CA125, and AFP level measurement), chest radiography, abdominal ultrasound or computed tomography, and annual endoscopy. OS was defined as the time from surgery date to death from any cause or to the date of the last follow-up. RFS was defined as the period from the first surgical treatment to the time of documented tumor recurrence (confirmed using imaging). The follow-up time of the entire cohort ranged from 5 to 133 months, with a median follow-up time of 62.5 months.

### Definition of trajectory groups

GBTM is primarily used to analyze longitudinal data to explore population heterogeneity. This approach assumes heterogeneity in the overall population, that is, the existence of several potential subgroups with distinct developmental trajectories or patterns within the population [33, 34, 41]. The trajectory model is selected and evaluated based on Bayesian Information Criterion (BIC). BIC is used to determine the number of trajectory groups. The optimal shape and parameters of the trajectory model (linear, quadratic, cubic, or higher) were tested using the maximum likelihood method [42]. For estimating the probability of each group member, each trajectory group’s probability should be ≥ 0.70, assigned to each trajectory group according to their average posterior probability (AvePP). In addition, the sample size of each group should include at least 5% of the included patients. The analytical method also includes a procedure for estimating missing data (at least three blood tests are required to ensure the correctness of grouping and the accuracy of parameter estimates) [43, 44]. GBTM is implemented using the traj plugin in STATA [45] (For a detailed explanation of GBTM, see Supplementary Materials). We assessed longitudinal trends in NLR during NACT using a multivariate latent classification model of the GBTM. GBTM identified unique trajectories by numerical change patterns at multiple time points and analyzed the differences between trajectory groups to explore the potential association between trajectory groups and the survival prognosis of GC.

### Statistical analysis

Statistical analysis was performed using SPSS software (version 24.0) and STATA version 15 (StataCorp, College Station, TX, USA). If the continuous variables are normally distributed, they are expressed as the mean ± standard deviation (SD); otherwise, they are expressed as the median (IQR). Categorical variables are expressed as percentiles. One-way analysis of variance (ANOVA) or Kruskal-Wallis H test was used for continuous variables, while the chi-square or Fish exact test was used for categorical variables. The ROC curve and the AUC were used to evaluate the prognostic efficacy of each inflammatory marker. The logistic regression and Cox proportional hazards models were used for univariate and multivariate analysis. Statistical significance was tested using the likelihood ratio test. In addition, we compared the hazard ratios (HR) or odds ratios (OR) and their 95% confidence intervals (CIs) between the trajectory groups. The OS and RFS of GC patients were analyzed using the Kaplan-Meier survival curve, and the differences were evaluated using the Log-rank test. A two-tailed *P* < 0.05 was considered statistically significant.

## Results

### Baseline characteristics

A total of 216 GC patients were finally included in this study. Comparing the ROC curves of traditional inflammatory markers for predicting 5-year OS and 5-year RFS, the NLR was finally screened out. The five blood sample detection points had good AUC values; therefore, they were used as representative inflammatory markers in this study (Supplementary Table S1-2). Table 1 shows the preoperative clinical characteristics of the overall cohort. The average age was 59.0 years (10.5). There were 161 male patients (74.5%) and 55 female patients (25.5%). In addition, 176 patients (81.5%) were in clinical stage III-IV. The median cycle of NACT was 3.0(2.0–4.0) cycles, and there were 28 cases (13.0%) with grade 3 or 4 adverse reactions during NACT (Table 1). Detailed adverse reactions are presented in Supplementary Table S3. The tumors were located in the upper, middle, and lower regions [113 cases (52.3%), 57 cases (26.4%), and 46 cases (21.3%), respectively]. Distal gastrectomy was performed in 68 patients (31.5%), and total gastrectomy was performed in 148 patients (48.5%). Tumor regression grade: complete tumor regression (grade 0) accounted for 4.6% of patients, and no tumor regression accounted for 51.3%. There was no significant difference in tumor regression grade among the three groups (*p* = 0.898). Postoperative pathological staging was in 69 patients (31.9%), and 111 patients (31.9%) were in stage III (Table 1). There were 57 patients (26.4%) with grades I-II and 11 (5.1%) with grades III-IV. All postoperative patients received adjuvant chemotherapy routinely. The median cycle was 3.0 (2.0–4.0) cycles (Table S1). Follow-up for the entire cohort ranged from 5 to 133 months, with a median follow-up of 62.5 months. Significant differences (*p* < 0.05) in the intraoperative blood loss occurred in the trajectory group. It is likely that the combined effects of neutrophilia and lymphocytopenia lead to a high NLR and thus promote angiogenesis and inhibit anti-tumor reactivity, ultimately promoting tumor growth and progression [46–48]. Therefore, the NLR level in the preoperative up-down group and the continuous declining group is higher than that in the stable group, causing tissue edema and congestion, which may lead to relatively more intraoperative blood loss.

**Table 1:** Baseline characteristics

Variable^a^	Total (*n* = 216)	Stable (*n* = 89)	Ascent-descend (*n* = 80)	Continuous descent (*n* = 47)	p value^b^
**Age,mean(SD)**	59(10.5)	60(10.9)	58(10.5)	59(9.7)	0.534
**Gender,No(%)**					**0.041**
Male	161(74.5)	60(67.4)	60(75.0)	41(87.2)	
Female	55(25.5)	29(32.6)	20(25.0)	6(12.8)	
**Body mass index,median (IQR)**	21.9(19.5–24.3)	21.7(19.1–24.1)	22.4(20.4–24.5)	21.4(19.3–24.5)	0.382*
**ECOG PS,No(%)**					0.772
0	128(59.3)	53(59.6)	49(61.3)	26(55.3)	
1/2	88(40.7)	36(40.4)	31(38.7)	21(44.7)	
**CT cT stage,No(%)**					0.619^#^
T2	3(1.4)	2(2.2)	0(0)	1(2.1)	
T3	45(20.8)	16(18.0)	18(22.5)	11(23.4)	
T4	168(77.8)	71(79.8)	62(77.5)	35(74.5)	
**CT cN stage,No(%)**					0.605
N0	37(17.1)	18(20.2)	12(15.0)	7(14.9)	
Nx	179(82.9)	71(79.8)	68(85.0)	40(85.1)	
**CT cTNM stage,No(%)**					0.639^#^
I	2(0.9)	1(1.1)	0(0)	1(2.1)	
II	38(17.6)	18(20.2)	12(15.0)	8(38.3)	
III-IV	176(81.5)	70(78.7)	68(85.0)	38(80.9)	
**No.of Cycle NAC completed(IQR)**	3(2–4)	3(2–4)	3(2–4)	3(2–4)	0.833
**Grade 3 or 4 adverse effects**^**c**^, **yes(%)**	28(13.0)	12(13.5)	10(12.5)	6(12.8)	0.981
**Resection range**					0.04
Distal		68(31.5)	36(40.4)	22(27.5)	10(21.3)
Total		148(68.5)	53(59.6)	58(72.5)	37(78.7)
**Multiorgan resection (yes)**	25(11.8)	11(12.4)	8(10.0)	6(12.8)	0.855
**Intraoperative blood loss,median (IQR), ml**	50(30.0-100)	50(30.0–80.0)	50(31.3–100)	100(50.0-150)	0.020*
**No. of lymph nodes dissected,mean(SD)**	36.8(13.6)	36.4(13.6)	35.3(11.6)	39.9(16.1)	0.170*
**No.of positive lymph nodes median (IQR)**	2.0(0.0–7.0)	1.0(0.0–4.0)	3.0(0.0-7.8)	3.0(0.0–9.0)	0.097*
**Tumor location**					0.550
Upper	113(52.3)	42(47.2)	45(56.2)	26(55.3)	
Middle	57(26.4)	24(27.0	19(23.8)	14(29.8)	
Lower	46(21.3)	23(25.8)	16(20.0)	7(14.9)	
**Lymphovascular invasion(yes)**	79(36.6)	28(31.5)	36(45.0)	17(36.2)	
**Neural infiltration(yes)**	28(31.5)	34(38.2)	34(42.5)	28(31.5)	
**Tumor regression grade**					0.898^#^
0	10(4.6)	4(4.5)	5(6.2)	1(2.1)	
1	33(15.3)	15(16.8)	12(15.0)	6(12.8)	
2	62(28.7)	28(31.5)	21(26.3)	13(27.7)	
3	111(51.3)	42(47.2)	42(52.5)	27(57.4)	
**ypT stage**					0.795^#^
ypT0	11(5.1)	5(5.6)	5(6.3)	1(2.1)	
ypT1	14(6.5)	5(5.6)	4(5.0)	5(10.6)	
ypT2	22(10.2)	10(11.2)	7(8.8)	5(10.6)	
ypT3	106(49.1)	48(53.9)	38(47.5)	20(42.6)	
ypT4a	55(25.4)	19(21.3)	23(28.7)	13(27.7)	
ypT4b	8(3.7)	2(2.2)	3(3.7)	3(6.4)	
**ypN stage**					0.198
ypN0	71(32.9)	30(33.7)	26(32.5)	15(31.9)	
ypN1	39(18.0)	23(25.8)	11(13.7)	5(10.6)	
ypN2	51(23.6)	19(21.4)	21(26.3)	11(23.4)	
ypN3	55(25.5)	17(19.1)	22(27.5)	16(34.1)	
**ypTNM stage**					0.615^#^
T0Nx	2(0.9)	0(0)	1(1.3)	1(2.1)	
I	34(15.8)	15(16.9)	12(15.0)	7(14.9)	
II	69(31.9)	33(37.1)	24(30.0)	12(25.5)	
III	111(51.4)	41(46.0)	43(53.7)	27(57.5)	
**Post-operative hospital stay (IQR), d**	9.0(7.0–12.0)	9.0(7.0–11.0)	9.0(7.0–12.0)	10.0(8.0–13.0)	0.952
**Time to first, d**					
Aerofluxus, mean (SD)	3.55(0.73)	3.42(0.77)	3.64(0.70)	3.64(0.71)	0.090
Walking activity,mean (SD)	2.59(1.80)	2.53(0.77)	2.78(2.82)	2.40(0.54)	0.277
Half-liquid die,mean (SD)	4.72(3.22)	4.67(4.17)	4.84(2.85)	4.60(1.06)	0.908
Pull out the drainage tube,mean (SD)	8.82(4.26)	8.99(5.59)	8.27(2.66)	9.43(3.418)	
**Postoperative blood transfusion (yes)**	21(9.7)	12(13.5)	4(5.0)	5(10.6)	0.167^#^
**Clavien-Dindo classification**					**0.005** ^**#**^
Grade I	22(10.2)	6(6.7)	11(13.8)	5(10.6)	
Grade II	35(16.2)	16(18.0)	6(7.5)	13(27.7)	
Grade III	8(3.7)	0(0)	5(6.3)	3(6.4)	
Grade IV	3(1.4)	1(1.1)	1(1.3)	1(2.1)	
Grade V	0(0)	0(0)	0(0)	0(0)	
**No. of completed** **chemotherapy cycles, median (IQR)**	3(2–4)	4(2–4)	3(2–3)	3(2–3)	0.174

*Note *^**a**^ Expressed as (%) if the variable is categorical, mean (SD) if continuous and normally distributed, median (IQR) if continuous and non-normal distributed

^**b**^ For continuous variables, if they are normally distributed, one-way analysis of variance (ANOVA) is used; otherwise, Kruskal-Wallis H test is used, which is represented by *. For categorical variables, the Chi-square test was used, with # indicating the accurate Fish test

^**c**^ For detailed adverse reactions, see Supplementary Materials

### Trajectory groups

**Fig. 1:**
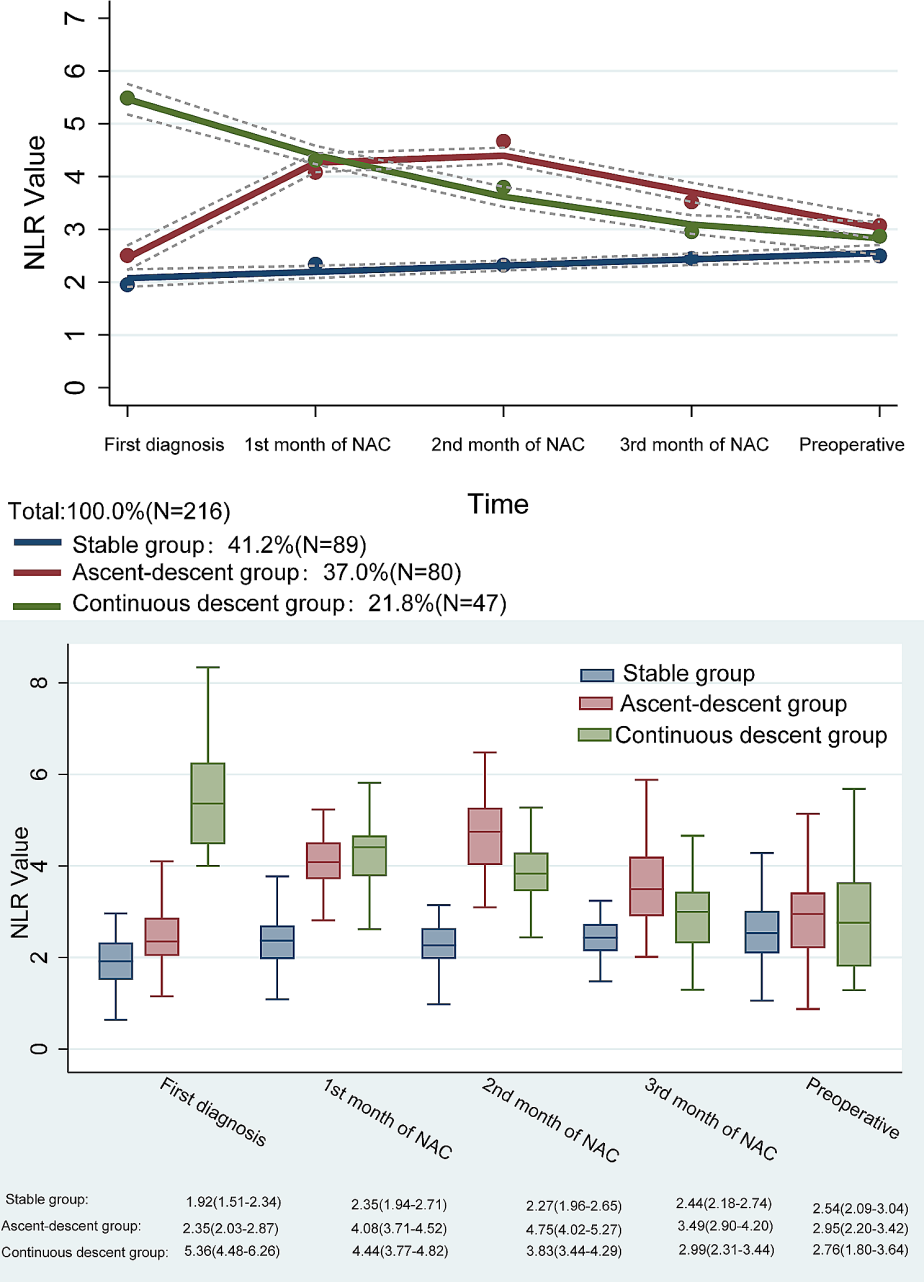
Group based trajectory model analysis of neutrophils and lymphocytes ratio in different track changes during neoadjuvant chemotherapy and values

The GBTM finally identified three distinct groups of NLR trajectories (Fig. 1). According to the change-trend in each trajectory group, they were named Stable Group (SG) (*n* = 89), Ascent- Descend Group (ADG) (*n* = 80) and Continuous Descend Group (CDG) (*n* = 47). The BIC value of the trajectory model is -1627.34, the linear term of SG is the best fit, the cubic term of ADG is the best fit, and the quadratic term of CDG is the best fit. The AvePP of the three groups was > 0.70 (Table S4-S6). The numerical visualization showed the changes in NLR values of the three trajectories at all time points to facilitate the cognition of the distribution of NLR values (Fig. 1). The NLR values of the three groups of patients at all time points were statistically significantly different (all *p* < 0.05). The median NLR of SG at the time of tumor diagnosis was 1.92(1.51–2.34), and the overall change trend during NACT was stable, with the highest median NLR of 2.54 (2.09–3.04). The median NLR of ADG at the time of tumor diagnosis was 2.35(2.03–2.87), and the overall trend of ADG during NACT was similar to a peak, with a peak median NLR of 4.75 (4.02–5.27). The median NLR of CDG at the time of tumor diagnosis was 5.36(4.48–6.26), and the NLR value continued to decrease during NACT, decreasing to 2.76 (1.80–3.64) at the preoperative time point (Fig. 1). Except for gender, intraoperative blood loss, and postoperative CDC grade, there were no significant differences in other clinicopathological characteristics (*p* > 0.05) (Table 1).

### Analysis of severe postoperative complications

Due to the significant difference in CDC grade among the three trajectory groups, the severe postoperative complications of CDG were significantly higher than those of SG and ADG [17(36.2%) vs. 17(19.1%) and 12(15.0%), *P* = 0.005] (Table 2). Incorporating this into further analysis, in logistic univariate and multivariate analyses, CDG was significantly associated with higher-grade postoperative complications (OR: 3.099; 95% CI: 1.325–7.250, *P* = 0.009)(Table S7). With SG as the reference group, the OR values of ADG and CDG were 0.747 (95% CI: 0.333–1.680; *p* = 0.481) and 2.400 (95% CI: 1.083–5.319; *p* = 0.031) (unadjusted), respectively. In addition, the adjusted OR values for ADG and CDG after fully adjusting the model were 0.820 (95% CI: 0.337–1.997; *p* = 0.663) and 3.660 (95% CI: 1.392–9.622; *p* = 0.009), respectively (Table 1). This indicated that CDG was more likely to develop severe postoperative complications compared with the other two trajectory groups. From the trajectory diagram (Fig. 1), it can be seen that the higher the NLR value at the time of the first tumor diagnosis, the higher the incidence of severe CDC grade after surgery. We conducted further analysis to investigate the potential correlation between NLR trajectories and clinical response to CT. The results, as presented in (Table S8), indicate that in both logistic univariate and multivariate analyses, there was no significant correlation between trajectory groups and CT clinical response (p-values all > 0.05). We also further analysis to investigate the potential association between NLR trajectories and adverse reactions to NAC. It indicates that in both logistic univariate and multivariate analyses, there was no significant correlation between trajectory groups and adverse reactions to NAC (p-values all > 0.05)(Table S9).

**Table 2:** Odds Ratio (OR) of serious postoperative complications in the trajectory groups

	Unadjusted model		Preliminary adjusted model^a^		Fully adjusted model^b^	
Higher CDC ratings	Odds Ratio (95% CI)	p value	Odds Ratio (95% CI)	p value	Odds Ratio (95% CI)	p value
Stable	Reference		Reference		Reference	
Ascent-descend	0.747(0.333–1.680)	0.481	0.818(0.358–1.870)	0.634	0.820(0.337–1.997)	0.663
Continuous descent	2.400(1.083–5.319)	0.031	2.565(1.123–5.862)	0.025	3.660(1.392–9.622)	0.009

*Note *^a^ Adjusted for age, gender, BMI, ECOG scoring scale

^b^ Additional adjusted for CT TNM stage, Tumor location, Resection range, Multiorgan resection (yes/no), Intraoperative blood loss, number of lymph node dissections, Lymphovascular invasion(yes/no), Neural infiltration (yes/no), Tumor regression grade, ypTNM stage

### Analysis of postoperative recurrence and mortality

**Fig. 2:**
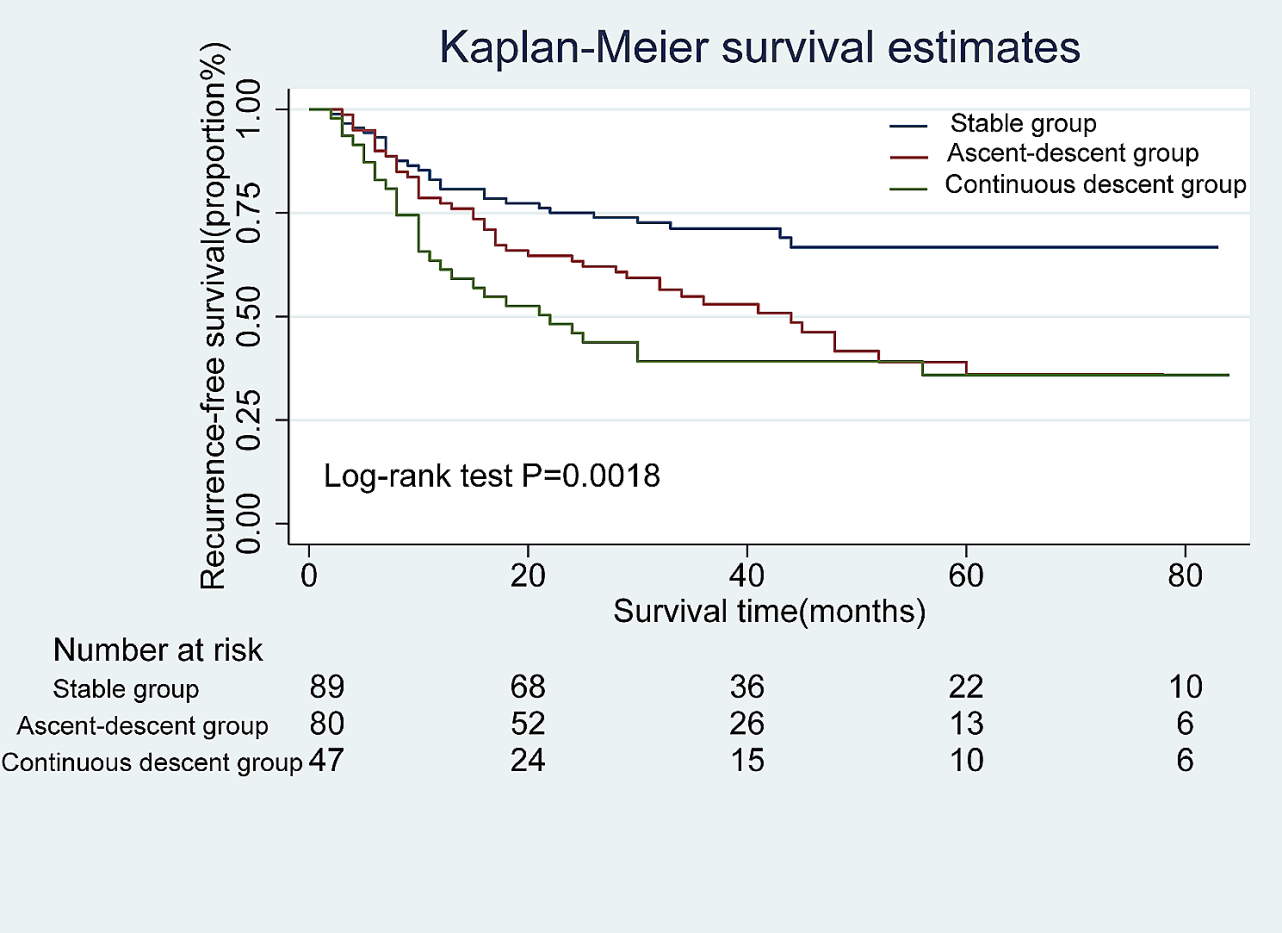
Kaplan-Meier survival curves for Recurrence-Free Survival for different trajectory groups

**Fig. 3:**
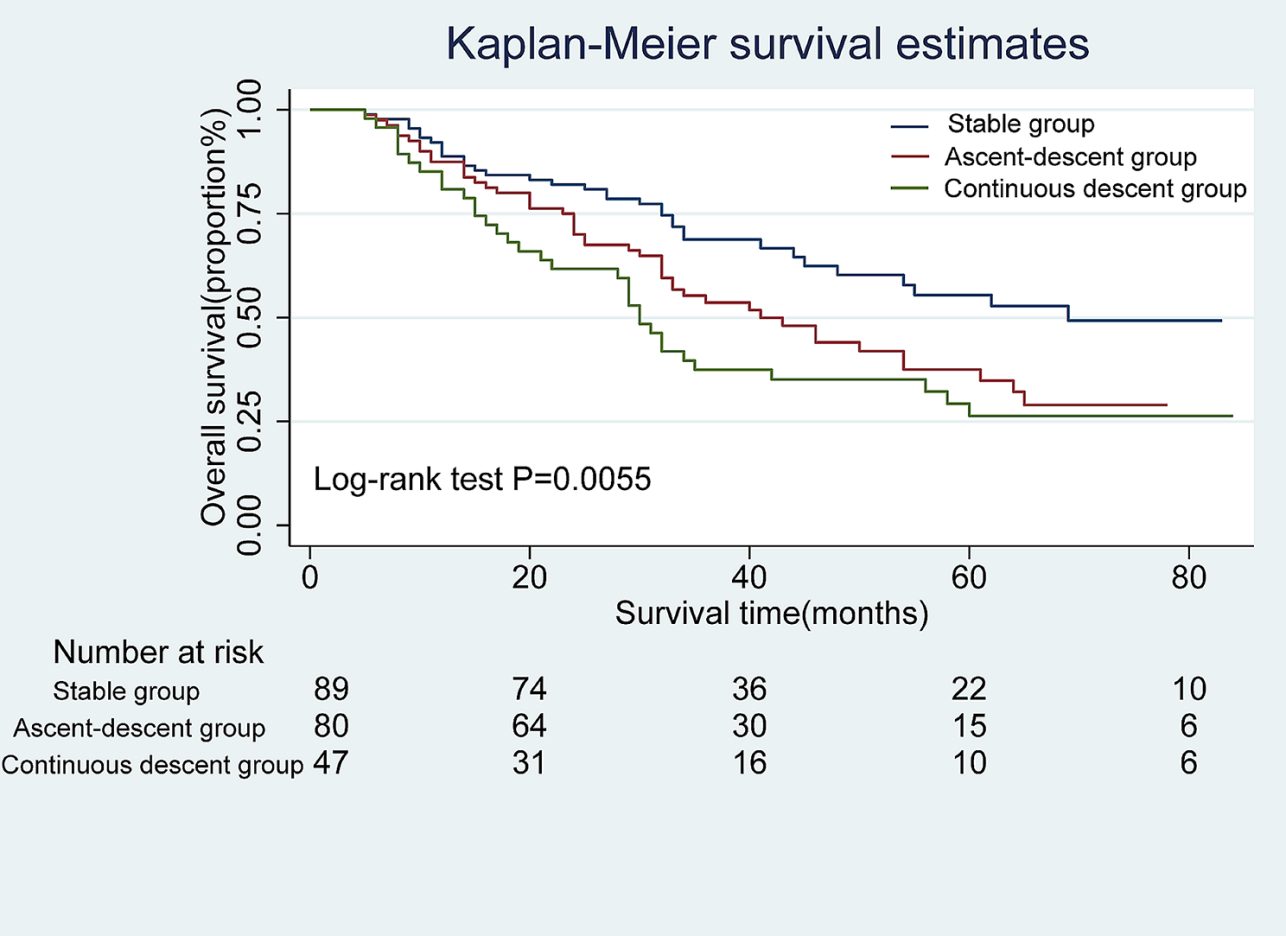
Kaplan-Meier survival curves for Overall Survival for different trajectory groups

Cox univariate and multivariate analyses were performed for RFS and OS. In univariate analysis, trajectory groups, number of positive lymph nodes, more advanced ypN stage, lymphovascular invasion, and more advanced ypTNM stage were associated with OS and RFS (all *P* < 0.05). In multivariate analysis, trajectory groups remained an independent risk factor for OS and RFS (all P values < 0.05) (Table S10-S11). With SG as the reference group, the risk of postoperative tumor recurrence was increased in other trajectory groups, and the HR values of ADG and CDG were 1.905(95% CI: 1.185–3.060; *P* = 0.008) and 2.400 (95% CI: 1.426–4.039; *P* = 0.001) (unadjusted), respectively. In addition, the adjusted HR values for ADG and CDG after fully adjusted model were 1.989 (95% CI: 1.157–3.419; *P* = 0.013) and 2.031 (95% CI: 1.098–3.757; *P* = 0.024), respectively (Table 3). Furthermore, by plotting the Kaplan-Meier survival curve of RFS of the trajectory groups, the results showed that the median recurrence time of ADG was 44 months, while that of CDG was shorter, only 22 months (Fig. 2). This indicates that compared with SG, the other two trajectory groups have a higher probability of postoperative tumor recurrence, especially CDG. Univariate and multivariate analyses showed that the trajectory groups were significantly correlated with OS after NACT (all *P* < 0.005)(Table S9). With SG as the reference group, the HR values for ADG and CDG were 1.621 (95% CI: 1.052–2.497; *p* = 0.029) and 2.088 (95% CI: 1.306–3.339; *p* = 0.002) (unadjusted), respectively. The HR values for ADG and CDG after fully adjusting the model were 1.830 (95% CI: 1.127–2.969; *p* = 0.014) and 1.913 (95% CI: 1.092–3.351; *p* = 0.023), respectively (Table 4). The Kaplan-Meier survival curve of OS showed that NLR had a large trend during NACT, and the OS time was shorter, with a median survival time of 41 months for ADG and 30 months for CDG (Fig. 3). Kaplan-Meier survival curves of both OS and RFS were significantly different (Log-rank test: *P* = 0.0018 and *P* = 0.0055) (Figs. 2 and 3). In this study, we grouped the preoperative neoadjuvant chemotherapy NLR values and preoperative NLR values in the stable group according to the X-tile software. At the first admission, the cut-off for overall survival (OS) and relapse-free survival (RFS) was 2.46. Based to the high NLR and low NLR, the survival in RFS or OS (Figure S2A and S2B). Similarly, we also analyzed the difference between high NLR or low LR groups in the stable group (OS: 2.36 and RFS: 2.30, respectively), and found no statistical difference in survival prognosis between the high NLR and low NLR groups in the stable group (Figure S2C and S2D). We assessed the prognostic accuracy of the trajectory prediction model using receiver-operator characteristic (ROC) analyses and the AUC value were 0.72,95%CI:63.44–81.49 (Figure S3).

**Table 3:** Hazard ratios (HRs) for tumor recurrence in the trajectory groups

	Unadjusted model		Preliminary adjusted model^a^		Fully adjusted model^b^	
RFS	Hazard Ratio (95% CI)	p value	Hazard Ratio (95% CI)	p value	Hazard Ratio (95% CI)	p value
Stable	Reference		Reference		Reference	
Ascent-descend	1.905 (1.185–3.060)	0.008	1.970 (1.221–3.178)	0.005	1.989 (1.157–3.419)	0.013
Continuous descent	2.400 (1.426–4.039)	0.001	2.322 (1.363–3.985)	0.002	2.031 (1.098–3.757)	0.024

*Note *^a^ Adjusted for age, gender, BMI, ECOG scoring scale

^b^ Additional adjusted for CT TNM stage, Clinical response per RECIST criteria, Tumor location, Resection range, Multiorgan resection (yes/no), Intraoperative blood loss, number of lymph node dissections, Lymphovascular invasion (yes/no), Neural infiltration (yes/no), Tumor regression grade, ypTNM stage

**Table 4:** Trajectory group hazard ratio (HR) for overall survival

	Unadjusted model		Preliminary adjusted model^a^		Fully adjusted model^b^	
OS	Hazard Ratio (95% CI)	p value	Hazard Ratio (95% CI)	p value	Hazard Ratio (95% CI)	p value
Stable	Reference		Reference		Reference	
Ascent-descend	1.621(1.052–2.497)	0.029	1.639(1.061–2.530)	0.026	1.830(1.127–2.969)	0.014
Continuous descent	2.088(1.306–3.339)	0.002	2.025(1.250–3.281)	0.004	1.913(1.092–3.351)	0.023

*Note *^a^ Adjusted for age, gender, BMI, ECOG scoring scale

^b^ Additional adjusted for CT TNM stage, Clinical response per RECIST criteria, Tumor location, Resection range, Multiorgan resection (yes/no), Intraoperative blood loss, number of lymph node dissections, Lymphovascular invasion (yes/no), Neural infiltration(yes/no), Tumor regression grade, ypTNM stage

## Discussion

In recent years, tumor-related inflammatory markers have become one of the new hotspots in tumor research. Inflammation and tumor growth are interdependent, and many studies have elucidated the role of systemic inflammatory factors in various cancers [16–19]. In addition, several studies have confirmed that preoperative or postoperative hematological indicators can effectively predict the long-term prognosis of GC patients [20–28]. However, no report has evaluated continuous longitudinal changes in inflammatory markers to predict tumor recurrence and long-term survival in GC. Considering that inflammatory markers may have varying degrees of influence on GC during NACT, it is hypothesized that sequential longitudinal changes in inflammatory markers during NACT may represent different clinical implications. In this study, we found that NLR was more strongly associated with 5-year recurrence and mortality by comparing the AUC values of multiple classical inflammatory markers for predicting long-term survival prognosis. We used GBTM to determine the developmental trajectory of the NLR in GC patients during NACT. We then assessed the impact of NLR on the prognosis of GC survival. There were three longitudinal patterns of NLR development during this period, characterized by stable, ascending-descending, and continuous descending. The risk of tumor recurrence and death was increased in ADG and CDG compared with SG. In addition, CDG had a greater risk of serious postoperative complications. The trajectory group was the most important variable for predicting survival. Compared with patients who did not experience large changes in NLR during NACT, those who experienced large increases or decreases in NLR values were at higher risk of poor prognosis. To our knowledge, this is the first longitudinal cohort study to explore related studies. The significance of an innovative research should not only have clinical significance for the research cancer, but also have research help for other related cancers, which is the research innovation of the article. In recent years, through the group based trajectory model (GBTM) research [29, 32], their results said trajectory model analysis to study the dynamic changes of a variety of cancer data to predict survival prognosis, so this study may be published to prompt other cancer related researchers related to inflammation markers, this may be some contribution to cancer research. Group based trajectory model (GBTM) limitations: the model assumes that the research object has the same trend, but this assumption is often not always meet, especially in the research center, the key research over time personal behavior, biomarkers or other phenomenon of interest trajectory, overall tend to have large heterogeneity. The larger the population data of this model, the better the trajectory simulation is. To solve this problem: some scholars have developed the combination of variable analysis and human-centered analysis, collectively referred to as Latent class model (LCM), it can identify homogeneity in greater heterogeneity group, according to the trajectory of heterogeneity in the group will overall divided into different potential categories, for further study the law of development within provide new ideas.

In the tumor microenvironment, increased neutrophils can secrete many reactive oxygen species, induce cellular DNA damage, trigger genetic instability, lead to tumogenesis, and promote tumor progression [49, 50]. As an important part of the body’s immune response, the lymphocyte population can enhance tumor immune surveillance and inhibit tumor cell proliferation, invasion, and metastasis [51, 52]. In trajectory analysis, relatively high initial NLR or preoperative NLR levels in advanced GC patients receiving NACT were associated with a poorer prognosis, which is consistent with other studies [26, 53–55]. It is worth noting that the development of the trajectory may reflect the developmental changes in the tumor or the response of the whole body after NACT, which may be the reason for the different trajectory trends. Although the two trajectory groups showed a greater reduction in NLR after NACT, there was no significant difference in NLR ratio change in the overall population after treatment, preoperative NLR, and pre-treatment NLR, which is similar to the results of Wang et al. [50]. Li et al. also studied the dynamic changes in inflammatory markers before GC. However, they were limited to two-time points, namely before chemotherapy and before surgery, and they achieved positive results. Furthermore, they may have ignored the different fluctuations of inflammatory factors during NACT and conducted a simple dichotomic analysis of the NLR values of patients with advanced GC [56]. After adjusting for multivariate factors, the different trajectories of NLR still had significant differences in the risk of prognostic survival, suggesting that it was an independent predictor of GC recurrence and death. Therefore, changes in inflammatory marker levels during NACT are of great significance in the preoperative monitoring of GC, and patients with large fluctuations in NLR during NACT should be closely monitored. This study has some limitations. First, as a single-center retrospective study, its results need to be further confirmed by large-sample, multicenter, prospective studies. Second, the patients included in the analysis had different NACT regimens, postoperative chemotherapy regimens, and non-identical chemotherapy cycles, which may have affected their survival prognosis to varying degrees. Third, the study results suggest that the Continuous Descend Group represents a highly malignant or inflammatory subgroup. Unfortunately, routine testing for C-reactive protein (CRP) levels at the initial hospital admission of gastric cancer patients was not conducted. Consequently, there is substantial missing data regarding this aspect. We acknowledge that this is a limitation of our study, as we are unable to fully depict the CRP levels at the time of initial diagnosis for each trajectory group. Regarding the investigation of CRP trajectories, we agree that it could be a novel avenue for future research. We plan to incorporate an analysis of dynamic changes in CRP levels in subsequent studies. The same regret is advanced gastric cancer patients for neoadjuvant chemotherapy and a series of preoperative evaluation before surgical treatment, so preoperative or intraoperative peritoneal cytology less in our center, so the lack of the data for deep analysis, this may be a very interesting innovation, after which we will be prospective data collection this data for follow-up analysis. Finally, the study population received NACT, no patients received radiotherapy or immune (targeted) therapy. Whether this conclusion can be generalized to this population remains to be further studied.

## Conclusion

In conclusion, the preoperative NLR of gastric cancer patients after NACT treatment can be divided into three clinical development patterns, and the trajectory development trend can predict the long-term prognosis and survival of GC. In addition, NLR potential trajectory is associated with postoperative complications. Therefore, a longitudinal trajectory can provide additional short-term and long-term curative effects of independent information and advice during NACT dynamic monitoring of NLR value to predict the clinical curative effect in patients with GC, using different trajectories for individualized treatment and follow-up strategies.

### Electronic supplementary material

Below is the link to the electronic supplementary material.

Supplementary Material 1: The inclusion and exclusion criteria and grouping of this study

## Data Availability

The dataset generated for this current study are not publicly available due additional research questions to be answered, but is available from the corresponding author on reasonable request.

## Abbreviations

GC: Gastric cancer
NACT: Neoadjuvant chemotherapy
ROC: Receiver operating characteristic
GBTM: Group-based trajectory modeling
RFS: Recurrence-free survival
OS: Overall survival
NLR: Neutrophil-lymphocyte ratio
AUC: Under the curve
SG: Stable Group
ADG: Ascent-descend group
CDG: Continuous descend group
PLR: Platelet-to-lymphocyte ratio
LMR: Lymphocyte-to-monocyte ratio
CRP/Alb: C-reactive protein/albumin
SII: Systemic immune inflammation index
mSIS: Modified systemic inflammation score
CI: Confidence interval
TRGs: Tumor regression grades
5-FU: 5-fluorouracil
XELOX/SOX: Oxaliplatin plus Capecitabine or S-1
CDC: Clavien-Dindo classification
BIC: Bayesian Information Criterion
AvePP: Average posterior probability
HR: Hazard ratios
OR: Odds ratios
95% CIs: 95% Confidence Intervals
