# Supplementary material for: Trajectories of neutrophil-to-lymphocyte ratios during neoadjuvant chemotherapy correlate with short- and long-term outcomes in gastric cancer: a group-based trajectory analysis
Source: BMC Cancer. 2024 Feb 16;24:226. doi: 10.1186/s12885-024-11950-2 (PMC10873962; doi:10.1186/s12885-024-11950-2)
Supplement: Supplementary file 1 — Supplementary Material 1: The inclusion and exclusion criteria and grouping of this study [file 12885_2024_11950_MOESM1_ESM.doc]

**Title: Trajectories of neutrophil-to-lymphocyte ratios during neoadjuvant chemotherapy correlate with short- and long-term outcomes in gastric cancer: a group-based trajectory analysis**

**Supplementary Data**

**Contents:**

1. Definition and calculation of inflammatory markers
2. Table.S1 AUC values (95% CI) of inflammatory markers for predicting 5-year recurrence-free survival in gastric cancer at 5 time points
3. Table.S2 AUC values (95%CI) of inflammatory markers for predicting 5-year overall survival in gastric cancer at 5 time points
4. Figure.S1 The inclusion and exclusion criteria and grouping of this study
5. Figure.S2 The first admission and preoperative NLR of the stable group were divided into low and low NLR group and high NLR
6. Figure. S3 The ROC curves for the trajectory survival predictive model
7. Table.S3 Specific circumstances in which grade 3 or 4 chemotherapy adverse reactions occurred
8. Definition of Group-Based Trajectory Model (GBTM)
9. Table.S4 Model selection results
10. Table.S5 Maximum Likelihood Estimates of the model parameters
11. Table.S6 Number of people assigned to each trajectory group based on average posterior probability (AvePP)
12. Table.S7 Univariate and multivariate logistic analysis of severe postoperative complications
13. Table.S8 Odds Ratio (OR) of CT clinical responses in the trajectory groups
14. Table.S9 Odds Ratio (OR) of the adverse effects by NACT in the trajectory groups
15. Table.S10 Univariate and multivariate cox regression analysis of recurrence-free survival
16. Table.S11 Univariate and multivariate cox regression analysis of overall survival

**Definition and calculation of inflammatory markers:**

NLR=Neutrophil-Lymphocyte Ratio

PLR=Platelet-Lymphocyte Ratio

NAR=Neutrophil-Albumin Ratio

PAR=Platelet-Albumin Ratio

LMR=Lymphocyte-Monocyte Ratio

Systemic Inflammation Score(SIS)=Preoperative Serum Albumin(Alb)/Lymphocyte- Monocyte Ratio (LMR)

Table. S1 AUC values (95% CI) of inflammatory markers for predicting 5-year recurrence-free survival in gastric cancer at 5 time points.

| **SIM** | **First diagnosis** | | **After diagnosis,month** | | | |
| --- | --- | --- | --- | --- | --- | --- |
|  |  |  | **1st** | **2nd** | **3rd** | **Preoperative** |
| **NLR** | **AUC** | **0.611** | **0.660** | **0.683** | **0.550** | **0.704** |
|  | 95%CI | 0.530-0.693 | 0.583-0.737 | 0.608-0.757 | 0.467-0.632 | 0.628-0.781 |
| **PLR** | **AUC** | **0.589** | **0.591** | **0.509** | **0.498** | **0.483** |
|  | 95%CI | 0.510-0.669 | 0.511-0.670 | 0.427-0.591 | 0.414-0.581 | 0.400-0.564 |
| **NAR** | **AUC** | **0.515** | **0.513** | **0.595** | **0.556** | **0.519** |
|  | 95%CI | 0.433-0.597 | 0.431-0.594 | 0.514-0.676 | 0.471-0.641 | 0.437-0.601 |
| **PAR** | **AUC** | **0.538** | **0.538** | **0.534** | **0.546** | **0.450** |
|  | 95%CI | 0.458-0.619 | 0.456-0.619 | 0.450-0.618 | 0.461-0.630 | 0.368-0.532 |
| **LMR** | **AUC** | **0.434** | **0.365** | **0.469** | **0.558** | **0.422** |
|  | 95%CI | 0.354-0.515 | 0.285-0.445 | 0.386-0.552 | 0.474-0.641 | 0.340-0.504 |
| **SIS** | **AUC** | **0.561** | **0.655** | **0.626** | **0.427** | **0.586** |
|  | 95%CI | 0.480-0.642 | 0.576-0.733 | 0.546-0.706 | 0.343-0.510 | 0.505-0.667 |

Note：NLR：Neutrophil-Lymphocyte Ratio；PLR：Platelet-Lymphocyte Ratio；NAR：Neutrophil-Albumin Ratio；PAR：Platelet-Albumin Ratio；LMR：Lymphocyte-Monocyte Ratio；SIS：Preoperative Serum Albumin(Alb)/Lymphocyte- Monocyte Ratio (LMR)

Table.S2 AUC values (95%CI) of inflammatory markers for predicting 5-year overall survival in gastric cancer at 5 time points.

| **SIM** | **First diagnosis** | | **After diagnosis,month** | | | |
| --- | --- | --- | --- | --- | --- | --- |
|  |  |  | **1st** | **2nd** | **3rd** | **Preoperative** |
| **NLR** | **AUC** | **0.571** | **0.657** | **0.715** | **0.533** | **0.713** |
|  | 95%CI | 0.490-0.652 | 0.581-0.733 | 0.644-0.787 | 0.451-0.615 | 0.641-0.785 |
| **PLR** | **AUC** | **0.548** | **0.547** | **0.469** | **0.441** | **0.445** |
|  | 95%CI | 0.467- 0.628 | 0.465-0.629 | 0.388-0.550 | 0.360-0.522 | 0.363-0.526 |
| **NAR** | **AUC** | **0.541** | **0.534** | **0.619** | **0.573** | **0.510** |
|  | 95%CI | 0.460-0.622 | 0.451-0.616 | 0.540-0.699 | 0.489-0.655 | 0.427-0.593 |
| **PAR** | **AUC** | **0.538** | **0.528** | **0.515** | **0.496** | **0.407** |
|  | 95%CI | 0.456-0.621 | 0.446-0.611 | 0.433-0.597 | 0.413-0.580 | 0.326-0.487 |
| **LMR** | **AUC** | **0.435** | **0.378** | **0.412** | **0.512** | **0.417** |
|  | 95%CI | 0.355-0.516 | 0.299-0.456 | 0.331-0.492 | 0.429-0.595 | 0.337-0.497 |
| **SIS** | **AUC** | **0.556** | **0.647** | **0.664** | **0.472** | **0.566** |
|  | 95%CI | 0.475-0.636 | 0.570-0.724 | 0.587-0.740 | 0.389-0.556 | 0.485-0.648 |

Note：NLR：Neutrophil-Lymphocyte Ratio；PLR：Platelet-Lymphocyte Ratio；NAR：Neutrophil-Albumin Ratio；PAR：Platelet-Albumin Ratio；LMR：Lymphocyte-Monocyte Ratio；SIS：Preoperative Serum Albumin(Alb)/Lymphocyte- Monocyte Ratio (LMR)

**Figure. S1.** The inclusion and exclusion criteria and grouping of this study


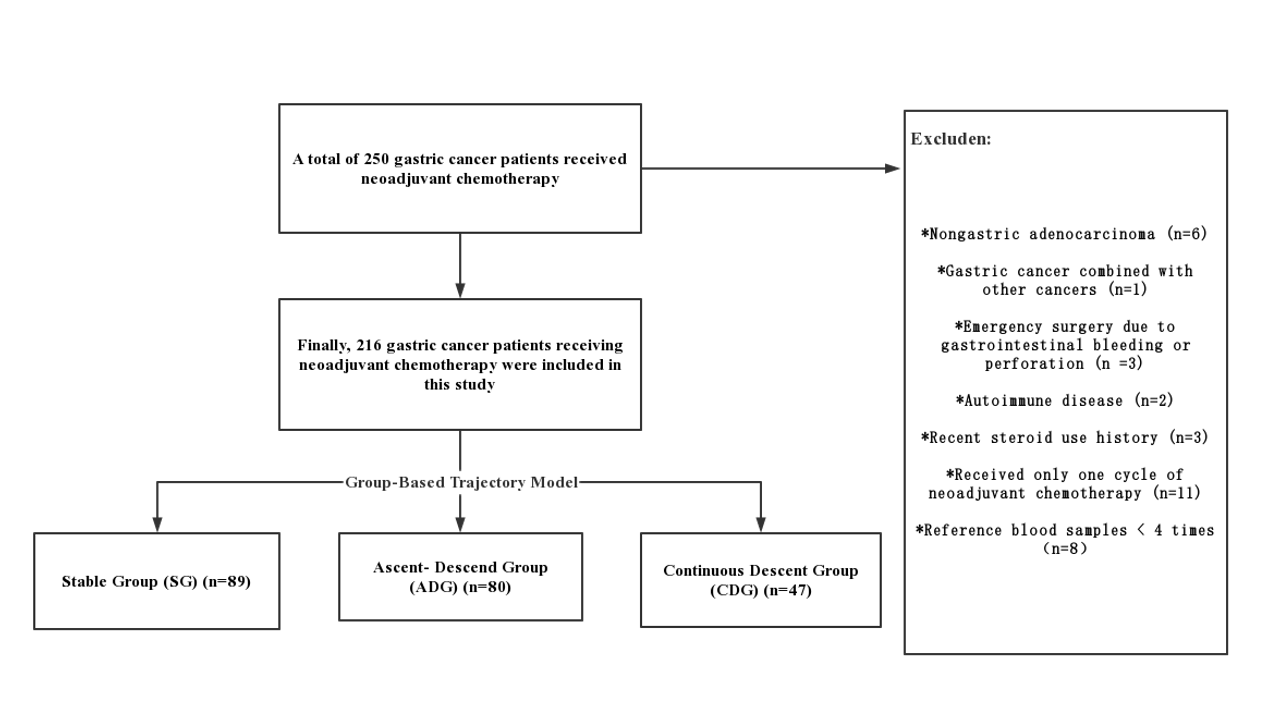


Figure S2：The first admission and preoperative NLR of the stable group were divided into low and low NLR group and high NLR.


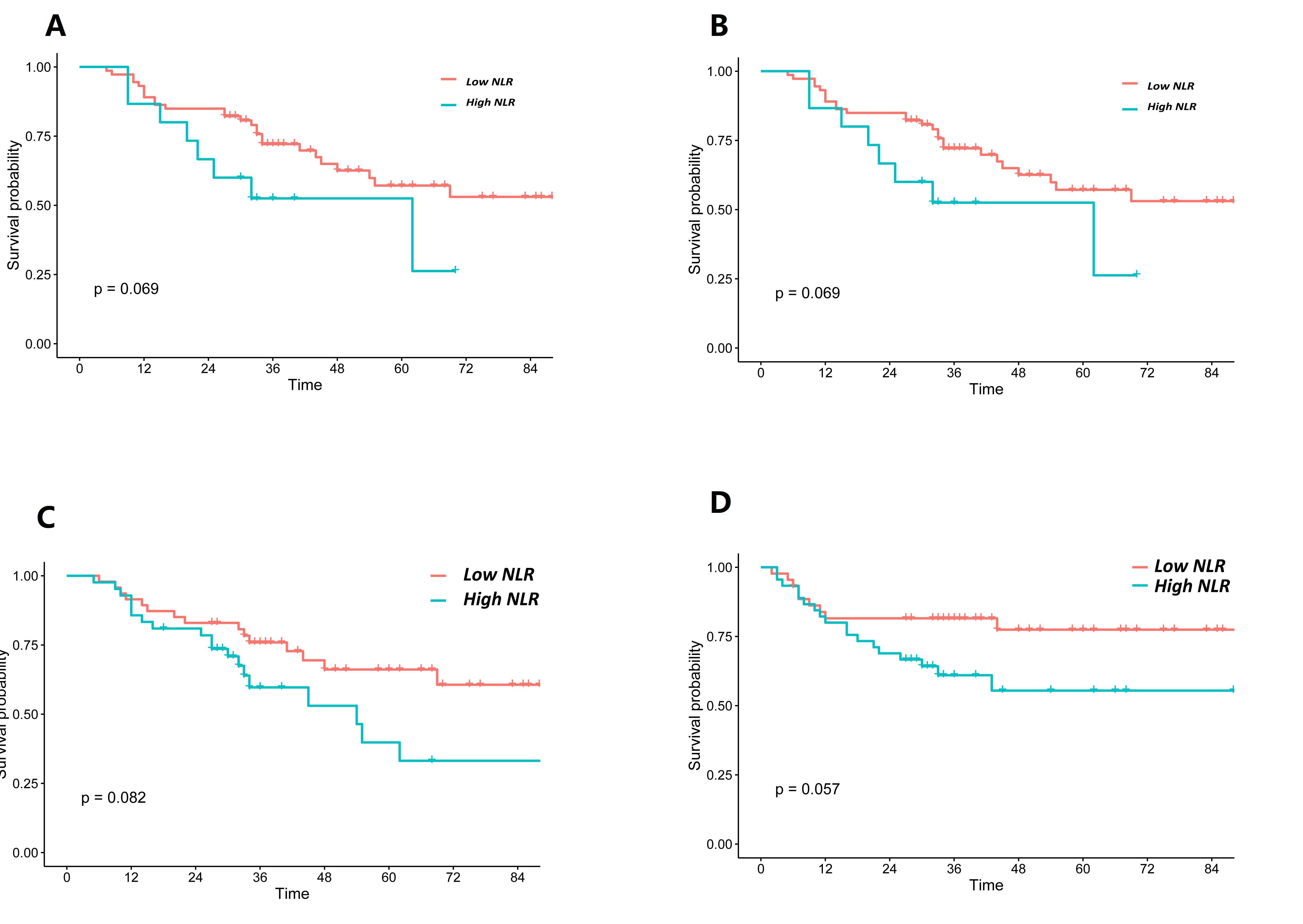


Figure. S3：The ROC curves for the trajectory survival predictive model.


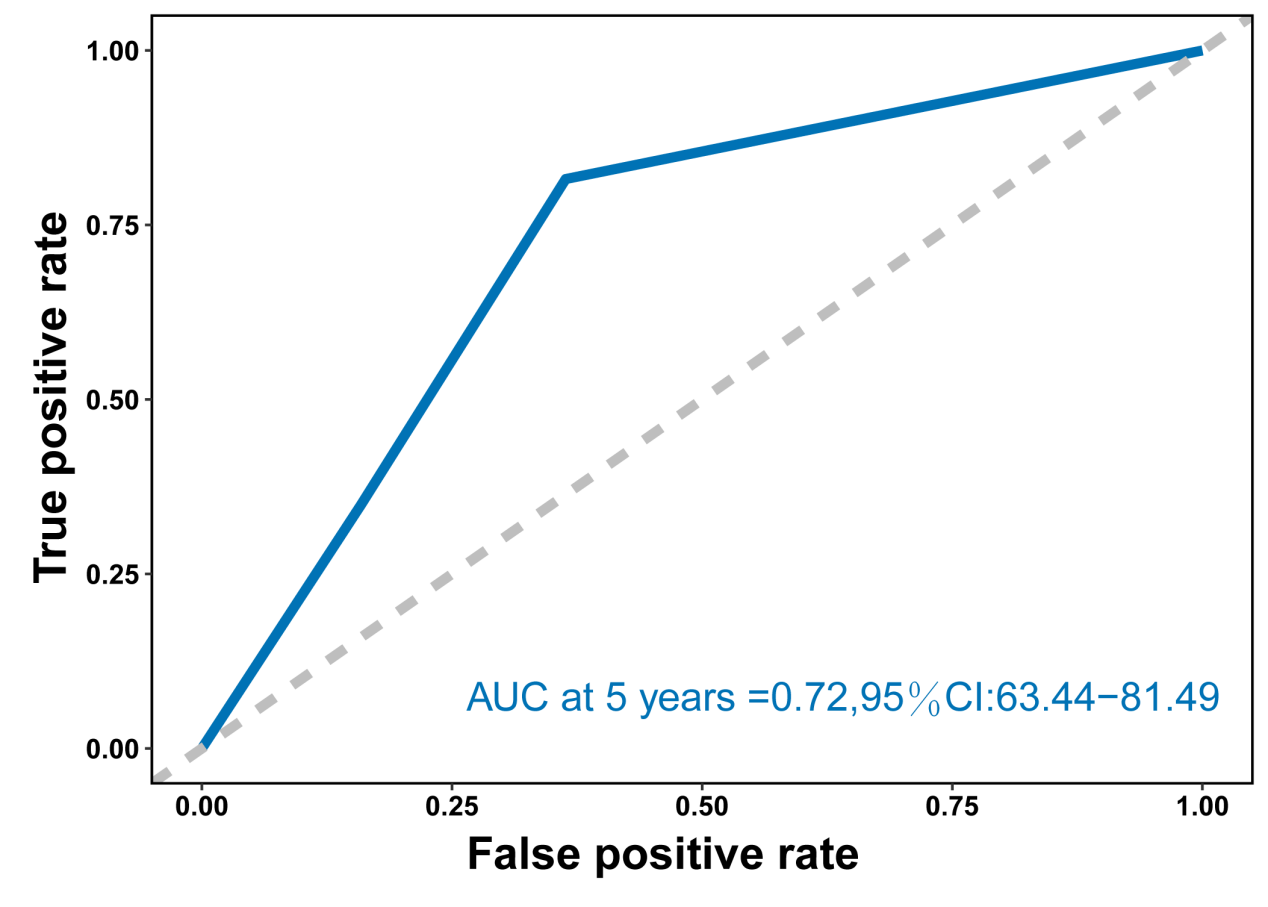


Table.S3 Specific circumstances in which grade 3 or 4 chemotherapy adverse reactions occurred

| **Grade 3 or 4 adverse effects (％)** | **Total(n=216)** | **Stable (n=89)** | **Ascent-descend (n=80)** | **Continuous descent (n=47)** |
| --- | --- | --- | --- | --- |
| White blood celldecrease | 4(1.9) | 1(1.1) | 2(2.5) | 1(2.1) |
| Neutrophil decrease | 8(3.7) | 4(4.5) | 3(3.8) | 1(2.1) |
| Anemia | 3(1.4) | 1(1.1) | 2(2.5) | 0(0.0) |
| Platelet decrease | 8(3.7) | 4(4.5) | 3(3.8) | 1(2.1) |
| Alanine aminotransferase increase | 1(0.5) | 1(1.1) | 0(0.0) | 0(0.0) |
| Total bilirubin increase | 1(0.5) | 1(1.1) | 0(0.0) | 0(0.0) |
| Fatigue | 0(0.0) | 0(0.0) | 0(0.0) | 0(0.0) |
| Anorexia | 1(0.5) | 0(0.0) | 0(0.0) | 1(2.1) |
| Difficulty swallowing | 0(0.0) | 0(0.0) | 0(0.0) | 0(0.0) |
| Stomach burning | 0(0.0) | 0(0.0) | 0(0.0) | 0(0.0) |
| Nausea or vomiting | 8(3.7) | 4(4.5) | 2(2.5) | 2(4.3) |
| Diarrhea | 1(0.5) | 0(0.0) | 1(1.3) | 0(0.0) |
| Itching | 4(1.9) | 2(2.2) | 2(2.5) | 0(0.0) |
| Neurotoxic effect | 2(0.9) | 0(0.0) | 1(1.3) | 1(2.1) |

**Definition of Group-Based Trajectory Model (GBTM)**

A potential classification model of the Group-Based Trajectory Model (GBTM) to assess longitudinal changes in neutrophil-to-lymphocyte ratio (NLR) in gastric cancer patients during NACT. GBTM describes the process by which NLR changes over time in CG patients during NACT, splitting the overall data into a number of trajectory groups and identifying longitudinal latent individuals who follow similar processes.Fit and test the overall data using different models. The details are as follows:

1. Estimating the number of trajectory group models

First, we perform model screening starting with a model consisting of a single trajectory group. Increase the number of trajectory groups until the most suitable trajectory model is determined.

Table.S4 Model selection results

| **Number of Trajectory Groups** | **Polynomial order** | **BIC** | **Bayes Factor*** | **Participants per group%** |
| --- | --- | --- | --- | --- |
| **1** | **3** | **-1783.26** | **0** | **100** |
| 1 | 2 | -1784.64 |  | 100 |
| 1 | 1 | -1801.06 |  | 100 |
| 2 | 3 3 | -1707.01 |  | 44.1/55.9 |
| 2 | 3 2 | -1710.82 |  | 44.6/55.4 |
| 2 | 3 1 | -1722.89 |  | 71.7/28.3 |
| 2 | 2 3 | -1705.02 |  | 43.9/56.1 |
| 2 | 2 2 | -1708.99 |  | 44.3/55.6 |
| 2 | 2 1 | -1724.33 |  | 70.2/29.8 |
| **2** | **1 3** | **-1704.09** | **79.17** | **42.9/57.1** |
| 2 | 1 2 | -1708.24 |  | 43.2/56.8 |
| 2 | 1 1 | -1736.12 |  | 47.5/52.5 |
| 3 | 3 3 3 | -1633.51 |  | 42.3/36.5/21.2 |
| 3 | 3 3 2 | -1630.75 |  | 42.3/37.0/20.7 |
| 3 | 3 3 1 | -1634 |  | 42.4/36.4/21.2 |
| 3 | 3 2 3 | -1642.16 |  | 42.5/36.3/21.2 |
| 3 | 3 2 2 | -1640.02 |  | 42.6/36.3/21.1 |
| 3 | 3 2 1 | -1644.67 |  | 42.7/36.1/21.2 |
| 3 | 3 1 3 | -1687.13 |  | 43.7/0.5/55.8 |
| 3 | 3 1 2 | -1690.27 |  | 44.2/0.5/55.3 |
| 3 | 3 1 1 | -1710.03 |  | 65.2/15.1/19.7 |
| 3 | 2 3 3 | -1631.53 |  | 42.2/36.7/21.1 |
| 3 | 2 3 2 | -1628.76 |  | 42.2/37.1/20.7 |
| 3 | 2 3 1 | -1631.99 |  | 42.3/36.5/21.2 |
| 3 | 2 2 3 | -1640.31 |  | 42.4/36.4/21.2 |
| 3 | 2 2 2 | -1638.18 |  | 42.4/36.5/21.1 |
| 3 | 2 2 1 | -1642.81 |  | 42.6/36.2/21.2 |
| 3 | 2 1 3 | -1685.17 |  | 43.5/0.5/56.0 |
| 3 | 2 1 2 | -1688.45 |  | 43.9/0.5/55.6 |
| 3 | 2 1 1 | -1710.49 |  | 58.1/23.9/18.0 |
| 3 | 1 3 3 | -1630 |  | 42.0/37.0/21.0 |
| **3*** | **1 3 2** | **-1627.34** | **76.75** | **41.9/37.5/20.6** |
| 3 | 1 3 1 | -1630.58 |  | 42.0/36.8/21.2 |
| 3 | 1 2 3 | -1638.8 |  | 42.1/36.8/21.1 |
| 3 | 1 2 2 | -1636.67 |  | 42.1/36.9/21.0 |
| 3 | 1 2 1 | -1641.41 |  | 42.3/36.6/21.1 |
| 3 | 1 1 3 | -1630.58 |  | 42.0/21.2/36.8 |
| 3 | 1 1 2 | -1687.58 |  | 43.0/0.5/56.5 |
| 3 | 1 1 1 | -1717.89 |  | 45.4/40.1/14.5 |
| **4** | **3 3 3 3** | **-1576.47** | **50.87** | **42.1/0.5/36.2/21.2** |
| 4 | 2 2 2 2 | -1590.67 |  | 42.3/0.5/36.3/20.9 |
| 4 | 1 1 1 1 | - |  | nonsymmetric or highly singular |

Note:Bayesian Information Criterion(BIC): The BIC is used to determine the optimal trajectory model in functional form. The closer the negative BIC value is to 0, the better the fit of the model.

Bayes Factor*: BIC2-BIC1. The BIC value between the two trajectory models with the smallest absolute value of BIC is at least greater than the difference value of 10 points, indicating that the trajectory model has a better model fit than the previous model, which is called the Bayes Factor*.

Table.S5 Maximum Likelihood Estimates of the model parameters.

| **Trajectory-group** | **Parameter order** | **Estimate** | **Standard Error** | **T value** | **Prob > |T|** |
| --- | --- | --- | --- | --- | --- |
| **Stable group** | Intercept | 2.0743 | 0.0763 | 27.2010 | 0.0000 |
|  | **Linear** | **0.1195** | **0.0303** | **3.9460** | **0.0001** |
| **Ascent-descend group** | Intercept | 2.4610 | 0.1141 | 21.5640 | 0.0000 |
|  | Linear | 2.9147 | 0.2703 | 10.7850 | 0.0000 |
|  | Quadratic | -1.2542 | 0.1656 | -7.5750 | 0.0000 |
|  | **Cubic** | **0.1402** | **0.0270** | **5.1930** | **0.0000** |
| **Continuous descent group** | Intercept | 5.4660 | 0.1476 | 37.0460 | 0.0000 |
|  | Linear | -1.1903 | 0.1666 | -7.1440 | 0.0000 |
|  | **Quadratic** | **0.1330** | **0.0402** | **3.3070** | **0.0010** |

Note: All retained polynomials in the trajectory group must maintain p-values < 0.05.

1. After trajectory model selection, we evaluate model adequacy based on the proportion of samples assigned to each identified trajectory group. This evaluation is based on the Average Posterior Probability (AvePP), ensuring that the individual AvePP assigned to each trajectory group is greater than a minimum threshold of 0.70 and based on the proportion of AvePP assigned to each identified trajectory group.

Table.S6 Number of people assigned to each trajectory group based on average posterior probability (AvePP)

|  | **AvePP - 1** | **AvePP - 2** | **AvePP - 3** | **OCC** | **Proportion assigned to group based on AvePP** | **N** |
| --- | --- | --- | --- | --- | --- | --- |
| **Stable group** | **0.97** | 0.02 | 0.01 | **46.1905** | **0.412** | **89** |
| **Ascent-descend group** | 0.02 | **0.96** | 0.02 | **42.8571** | **0.370** | **80** |
| **Continuous descent group** | 0.01 | 0.07 | **0.92** | **44.3331** | **0.218** | **47** |

Note:AvePP indicates average posterior probability of trajectory group membership; OCC, odds of correct classification calculated as [AvePP_i_/(1-AvePPi)] / [p_i_/(1-p_i_)]

Table.S7 Univariate and multivariate logistic analysis of severe postoperative complications

|  | Univariate analysis |  |  | Multivariate analysis |  |  |
| --- | --- | --- | --- | --- | --- | --- |
| **Variable** | OR | 95%CI | p value | OR | 95%CI | p value |
| **Trajectory groups** |  |  |  |  |  |  |
| Stable | Reference |  |  | Reference |  |  |
| Ascent-descend | 0.747 | 0.333-1.680 | 0.481 | 0.717 | 0.312-1.651 | 0.435 |
| Continuous descent | 2.400 | 1.083-5.319 | **0.031** | 3.099 | 1.325-7.250 | **0.009** |
| **Age** | 1.023 | 0.989-1.058 | 0.184 |  |  |  |
| **Gender** |  |  |  |  |  |  |
| Male | Reference |  |  |  |  |  |
| Female | 0.656 | 0.294-1.464 | 0.303 |  |  |  |
| **BMI** | 0.954 | 0.855-1.063 | 0.392 |  |  |  |
| **Neoadjuvant chemotherapy cycle** | 0.495 | 0.264-0.927 | 0.028 | 0.571 | 0.291-1.122 | 0.104 |
| **Adverse effects** | 0.318 | 0.385-18.959 | 0.318 |  |  |  |
| **ECOG PS** |  |  |  |  |  |  |
| 0 | Reference |  |  |  |  |  |
| 1/2 | 0.817 | 0.418-1.600 | 0.556 |  |  |  |
| **CT cT stage** |  |  |  |  |  |  |
| T2/T3 | Reference |  |  |  |  |  |
| T4 | 0.885 | 0.410-1.910 | 0.756 |  |  |  |
| **CT cN stage** |  |  |  |  |  |  |
| N0 | Reference |  |  |  |  |  |
| Nx | 0.680 | 0.302-1.532 | 0.352 |  |  |  |
| **CT cTNM stage** |  |  |  |  |  |  |
| I-II | Reference |  |  |  |  |  |
| III-IV | 0.559 | 0.258-1.210 | 0.140 |  |  |  |
| **Resection range** |  |  |  |  |  |  |
| Distal | Reference |  |  |  |  |  |
| Total | 1.605 | 0.759-3.393 | 0.215 |  |  |  |
| **Multiorgan resection** |  |  |  |  |  |  |
| No | Reference |  |  |  |  |  |
| Yes | 1.895 | 0.761-4.718 | 0.170 |  |  |  |
| **Intraoperative blood loss** | 1.000 | 0.997-1.003 | 0.985 |  |  |  |
| **No. of lymph nodes dissected** | 0.948 | 0.917-0.980 | **0.001** | 0.940 | 0.908-0.973 | **0.001** |
| **No.of positive lymph nodes** | 1.011 | 0.964-1.060 | 0.653 |  |  |  |
| **Tumor location** |  |  |  |  |  |  |
| Upper | Reference |  |  |  |  |  |
| Middle | 0.693 | 0.317-1.513 | 0.357 |  |  |  |
| Lower | 0.434 | 0.167-1.131 | 0.088 |  |  |  |
| **Lymphovascular invasion** |  |  |  |  |  |  |
| No | Reference |  |  |  |  |  |
| Yes | 1.290 | 0.663-2.511 | 0.423 |  |  |  |
| **Neural infiltration** |  |  |  |  |  |  |
| No | Reference |  |  |  |  |  |
| Yes | 0.901 | 0.460-1.767 | 0.762 |  |  |  |
| **Tumor regression grade** |  |  |  |  |  |  |
| 0 | Reference |  |  |  |  |  |
| 1 | 1.280 | 0.224-7.308 | 0.781 |  |  |  |
| 2 | 2.200 | 0.429-11.279 | 0.344 |  |  |  |
| 3 | 2.000 | 0.404-9.896 | 0.396 |  |  |  |
| **ypT stage** |  |  |  |  |  |  |
| ypT0 | Reference |  |  |  |  |  |
| ypT1 | 1.667 | 0.131-  21.195 | 0.694 |  |  |  |
| ypT2 | 4.667 | 0.495-  43.962 | 0.178 |  |  |  |
| ypT3 | 2.184 | 0.264-  18.098 | 0.469 |  |  |  |
| ypT4a | 4.103 | 0.484-  34.744 | 0.195 |  |  |  |
| ypT4b | 1.429 | 0.076-  26.895 | 0.812 |  |  |  |
| **ypN stage** |  |  |  |  |  |  |
| ypN0 | Reference |  |  |  |  |  |
| ypN1 | 0.723 | 0.235-2.228 | 0.572 |  |  |  |
| ypN2 | 1.860 | 0.776-4.457 | 0.164 |  |  |  |
| ypN3 | 1.844 | 0.781-4.351 | 0.163 |  |  |  |
| **ypTNM stage** |  |  |  |  |  |  |
| T0Nx-I | Reference |  |  |  |  |  |
| II | 0.702 | 0.242-2.033 | 0.515 |  |  |  |
| III | 1.465 | 0.579-3.705 | 0.420 |  |  |  |

Table.S8 Odds Ratio (OR) of CT clinical responses in the trajectory groups

|  | Unadjusted Model | | Preliminary Adjusted Model^a^ | | Fully adjusted model^b^ | |
| --- | --- | --- | --- | --- | --- | --- |
| CT clinical responses | Odds Ratio  (95% CI) | p value | Odds Ratio  (95% CI) | p value | Odds Ratio  (95% CI) | p value |
| Stable | Reference |  | Reference |  | Reference |  |
| Ascent-descend | 1.375(0.750-2.523) | 0.303 | 1.432(0.764-2.651) | 0.267 | 1.277(0.638-2.558) | 0.490 |
| Continuous descent | 1.444(0.708-2.944) | 0.312 | 1.605(0.773-3.332) | 0.204 | 1.373(0.587-3.210) | 0.465 |

Note: a: Adjusted for age, gender, BMI, ECOG scoring scale

b: Additional adjusted for Tumor location, Resection range, Multiorgan resection (yes/no), Intraoperative blood loss, number of lymph node dissections, Lymphovascular invasion(yes/no), Neural infiltration(yes/no), Tumor regression grade, ypTNM stage, postoperative complications

Table.S9 Odds Ratio (OR) of the adverse effects by NACT in the trajectory groups

|  | Unadjusted Model | | Preliminary Adjusted Model^a^ | | Fully adjusted model^b^ | |
| --- | --- | --- | --- | --- | --- | --- |
| Adverse Effects^c^ | Odds Ratio  (95% CI) | p value | Odds Ratio  (95% CI) | p value | Odds Ratio  (95% CI) | p value |
| Stable | Reference |  | Reference |  | Reference |  |
| Ascent-descend | 0.917(0.373-2.253) | 0.850 | 1.002(0.399-2.512) | 0.915 | 1.277(0.343-2.441) | 0.858 |
| Continuous descent | 0.939(0.328-2.685) | 0.907 | 0.922(0.314-2.711) | 0.883 | 1.245(0.380-4.078) | 0.718 |

Note: a: Adjusted for age, gender, BMI, ECOG scoring scale

b: Additional adjusted for CT TNM stage,Tumor location, Resection range, Multiorgan resection (yes/no), Intraoperative blood loss, number of lymph node dissections, Lymphovascular invasion(yes/no), Neural infiltration(yes/no), Tumor regression grade, ypTNM stage, postoperative complications

c: The adverse effects by NACT

Table.S8 Univariate and multivariate cox regression analysis of recurrence-free survival

|  | Univariate analysis |  |  | Multivariate analysis |  |  |
| --- | --- | --- | --- | --- | --- | --- |
| **Variable** | HR | 95%CI | p value | HR | 95%CI | p value |
| **Trajectory groups** |  |  |  |  |  |  |
| Stable | Reference |  |  | Reference |  |  |
| Ascent-descend | 1.905 | 1.185-3.060 | **0.008** | 1.730 | 1.066-2.806 | **0.026** |
| Continuous descent | 2.4 | 1.426-4.039 | **0.001** | 2.207 | 1.283-3.796 | **0.004** |
| **Age** | 0.995 | 0.977-1.013 | 0.552 |  |  |  |
| **Gender** |  |  |  |  |  |  |
| Male | Reference |  |  |  |  |  |
| Female | 0.826 | 0.522-1.307 | 0.414 |  |  |  |
| **BMI** | 0.948 | 0.888-1.012 | 0.109 |  |  |  |
| **Neoadjuvant chemotherapy cycle** | 0.693 | 0.470-1.022 | 0.064 |  |  |  |
| **Adverse effects** | 0.931 | 0.509-1.702 | 0.816 |  |  |  |
| **ECOG PS** |  |  |  |  |  |  |
| 0 | Reference |  |  |  |  |  |
| 1/2 | 1.147 | 0.773-1.702 | 0.497 |  |  |  |
| **CT cT stage** |  |  |  |  |  |  |
| T2/T3 | Reference |  |  |  |  |  |
| T4 | 1.106 | 0.694-1.763 | 0.672 |  |  |  |
| **CT cN stage** |  |  |  |  |  |  |
| N0 | Reference |  |  |  |  |  |
| Nx | 1.621 | 0.905-2.904 | 0.104 |  |  |  |
| **CT cTNM stage** |  |  |  |  |  |  |
| I-II | Reference |  |  |  |  |  |
| III-IV | 1.465 | 0.846-2.537 | 0.172 |  |  |  |
| **Resection range** |  |  |  |  |  |  |
| Distal | Reference |  |  |  |  |  |
| Total | 1.236 | 0.803-1.902 | 0.336 |  |  |  |
| **Multiorgan resection** |  |  |  |  |  |  |
| No | Reference |  |  |  |  |  |
| Yes | 0.858 | 0.445-1.652 | 0.646 |  |  |  |
| **Intraoperative blood loss** | 1.001 | 0.999-1.003 | 0.177 |  |  |  |
| **No. of lymph nodes dissected** | 1.007 | 0.992-1.021 | 0.385 | Reference |  |  |
| **No.of positive lymph nodes** | 1.100 | 1.075-1.127 | **0.001** | 1.054 | 1.007-1.103 | **0.025** |
| **Tumor location** |  |  |  |  |  |  |
| Upper | Reference |  |  |  |  |  |
| Middle | 1.233 | 0.784-1.940 | 0.364 |  |  |  |
| Lower | 0.874 | 0.521-1.467 | 0.610 |  |  |  |
| **Lymphovascular invasion** |  |  |  |  |  |  |
| No | Reference |  |  | Reference |  |  |
| Yes | 2.257 | 1.525-3.342 | **0.001** | 1.280 | 0.813-2.015 | 0.287 |
| **Neural infiltration** |  |  |  |  |  |  |
| No | Reference |  |  |  |  |  |
| Yes | 1.404 | 0.947-2.083 | 0.091 |  |  |  |
| **Tumor regression grade** |  |  |  |  |  |  |
| 0 | Reference |  |  |  |  |  |
| 1 | 0.510 | 0.149-1.746 | 0.284 |  |  |  |
| 2 | 2.006 | 0.713-5.643 | 0.187 |  |  |  |
| 3 | 1.500 | 0.543-4.146 | 0.434 |  |  |  |
| **ypT stage** |  |  |  |  |  |  |
| ypT0 | Reference |  |  |  |  |  |
| ypT1 | 0.564 | 0.126-2.527 | 0.454 |  |  |  |
| ypT2 | 1.052 | 0.308-3.595 | 0.935 |  |  |  |
| ypT3 | 1.683 | 0.609-4.656 | 0.316 |  |  |  |
| ypT4a | 2.178 | 0.768-6.178 | 0.143 |  |  |  |
| ypT4b | 2.246 | 0.560-9.004 | 0.253 |  |  |  |
| **ypN stage** |  |  |  |  |  |  |
| ypN0 | Reference |  |  | Reference |  |  |
| ypN1 | 1.371 | 0.654-2.876 | 0.403 | 1.174 | 0.510-2.705 | 0.706 |
| ypN2 | 3.270 | 1.814-5.894 | **0.001** | 1.504 | 0.609-3.787 | 0.376 |
| ypN3 | 5.858 | 3.295-  10.414 | **0.001** | 1.525 | 0.509-4.563 | 0.451 |
| **ypTNM stage** |  |  |  |  |  |  |
| T0Nx-I | Reference |  |  | Reference |  |  |
| II | 0.954 | 0.436-2.084 | 0.905 | 0.979 | 0.446-2.150 | 0.958 |
| III | 3.737 | 1.926-7.251 | **0.001** | 2.530 | 1.235-5.184 | **0.011** |
| **Clavien-Dindo classification** |  |  |  |  |  |  |
| Grade I-Grade II | Reference |  |  | Reference |  |  |
| Grade III-Grade IV | 1.530 | 1.026-2.281 | **0.037** | 1.156 | 0.724-1.845 | 0.545 |
| **Post-operative chemotherapy cycle** | 0.994 | 0.854-1.158 | 0.943 |  |  |  |

Table.S9 Univariate and multivariate cox regression analysis of overall survival

|  | Univariate analysis |  |  | Multivariate analysis |  |  |
| --- | --- | --- | --- | --- | --- | --- |
| **Variable** | HR | 95%CI | p value | HR | 95%CI | p value |
| **Trajectory groups** |  |  |  |  |  |  |
| Stable group | Reference |  |  | Reference |  |  |
| Ascent-descend | 1.621 | 1.052-2.497 | **0.029** | 1.574 | 1.019-2.430 | **0.041** |
| Continuous descent | 2.088 | 1.306-3.339 | **0.002** | 2.067 | 1.285-3.325 | **0.003** |
| **Age** | 1.008 | 0.990-1.026 | 0.394 |  |  |  |
| **Gender** |  |  |  |  |  |  |
| Male | Reference |  |  |  |  |  |
| Female | 0.837 | 0.547-1.279 | 0.411 |  |  |  |
| **BMI** | 0.988 | 0.930-1.050 | 0.699 |  |  |  |
| **Neoadjuvant chemotherapy cycle** | 0.728 | 0.503-1.054 | 0.092 |  |  |  |
| **Adverse effects** | 1.091 | 0.633-1.878 | 0.754 |  |  |  |
| **ECOG PS** |  |  |  |  |  |  |
| 0 | Reference |  |  |  |  |  |
| 1/2 | 1.407 | 0.979-2.022 | 0.065 |  |  |  |
| **CT cT stage** |  |  |  |  |  |  |
| T2/T3 | Reference |  |  |  |  |  |
| T4 | 1.114 | 0.724-1.715 | 0.622 |  |  |  |
| **CT cN stage** |  |  |  |  |  |  |
| N0 | Reference |  |  |  |  |  |
| Nx | 1.604 | 0.933-2.759 | 0.088 |  |  |  |
| **CT cTNM stage** |  |  |  |  |  |  |
| I-II | Reference |  |  |  |  |  |
| III-IV | 1.486 | 0.888-2.486 | 0.131 |  |  |  |
| **Resection range** |  |  |  |  |  |  |
| Distal | Reference |  |  |  |  |  |
| Total | 1.104 | 0.746-1.634 | 0.620 |  |  |  |
| **Multiorgan resection** |  |  |  |  |  |  |
| No | Reference |  |  |  |  |  |
| Yes | 1.168 | 0.696-1.958 | 0.557 |  |  |  |
| **Intraoperative blood loss** | 1.001 | 0.999-1.003 | 0.297 |  |  |  |
| **No. of lymph nodes dissected** | 1.005 | 0.991-1.020 | 0.448 |  |  |  |
| **No.of positive lymph nodes** | 1.094 | 1.070-1.119 | **0.001** | 1.068 | 1.039-1.098 | **0.002** |
| **Tumor location** |  |  |  |  |  |  |
| Upper | Reference |  |  |  |  |  |
| Middle | 1.237 | 0.810-1.890 | 0.326 |  |  |  |
| Lower | 0.950 | 0.595-1.517 | 0.830 |  |  |  |
| **Lymphovascular invasion** |  |  |  |  |  |  |
| No | Reference |  |  | Reference |  |  |
| Yes | 1.743 | 1.210-2.511 | **0.003** | 0.983 | 0.641-1.508 | 0.938 |
| **Neural infiltration** |  |  |  |  |  |  |
| No | Reference |  |  |  |  |  |
| Yes | 1.143 | 0.789-1.657 | 0.480 |  |  |  |
| **Tumor regression grade** |  |  |  |  |  |  |
| 0 | Reference |  |  |  |  |  |
| 1 | 0.792 | 0.252-2.489 | 0.689 |  |  |  |
| 2 | 2.203 | 0.787-6.171 | 0.133 |  |  |  |
| 3 | 1.712 | 0.623-4.706 | 0.297 |  |  |  |
| **ypT stage** |  |  |  |  |  |  |
| ypT0 | Reference |  |  |  |  |  |
| ypT1 | 0.756 | 0.189-3.027 | 0.692 |  |  |  |
| ypT2 | 1.601 | 0.502-5.108 | 0.426 |  |  |  |
| ypT3 | 1.848 | 0.670-5.100 | 0.236 |  |  |  |
| ypT4a | 1.824 | 0.989-3.364 | 0.054 |  |  |  |
| ypT4b | 2.590 | 0.693-9.682 | 0.157 |  |  |  |
| **ypN stage** |  |  |  |  |  |  |
| ypN0 | Reference |  |  | Reference |  |  |
| ypN1 | 1.317 | 0.706-2.454 | 0.386 | 1.134 | 0.789-2.778 | 0.222 |
| ypN2 | 2.348 | 1.392-3.962 | **0.001** | 1.283 | 0.638-3.787 | **0.001** |
| ypN3 | 4.501 | 2.707-7.483 | **0.001** | 2.382 | 1.107-5.128 | **0.001** |
| **ypTNM stage** |  |  |  |  |  |  |
| T0Nx-I | Reference |  |  | Reference |  |  |
| II | 0.907 | 0.464-1.774 | 0.775 | 0.961 | 0.466-1.802 | 0.800 |
| III | 2.949 | 1.669-5.209 | **0.001** | 1.983 | 1.066-3.687 | **0.031** |
| **Clavien-Dindo classification** |  |  |  |  |  |  |
| Grade I-Grade II | Reference |  |  |  |  |  |
| Grade III-Grade IV | 1.123 | 0.730-1.728 | 0.596 |  |  |  |
| **Post-operative chemotherapy cycle** | 0.992 | 0.861-1.143 | 0.913 |  |  |  |
